# Natural clay-supported palladium catalysts for methane oxidation reaction: effect of alloying†

**DOI:** 10.1039/c9ra06804j

**Published:** 2019-10-15

**Authors:** Yahia H. Ahmad, Assem T. Mohamed, Khaled A. Mahmoud, Amina S. Aljaber, Siham Y. Al-Qaradawi

**Affiliations:** Department of Chemistry and Earth Sciences, College of Arts and Sciences, Qatar University Doha 2713 Qatar siham@qu.edu.qa; Qatar Environment and Energy Research Institute (QEERI), Hamad Bin Khalifa University (HBKU) Doha 5825 Qatar

## Abstract

The catalytic combustion of methane (CCM) has been extensively studied owing to the wide use of methane in motor vehicles and power generation turbines. However, the absence of polarizability and the high C–H bond strength are considered to be the main drawbacks that limit its oxidation by traditional catalytic converters. Palladium-based catalysts are recognized as the benchmark catalysts for methane oxidation, especially under oxidizing conditions, and their activity is dependent on different parameters such as size, dispersion, and the nature of the support. Additionally, metal oxides are the most common supports used for CCM; however, they can become saturated with water, especially during steady-state operation at low temperatures, owing to their hydrophilic nature. This causes saturation of the active sites with OH species, which poisons the active centers of the catalyst, prevents activation of methane molecules, and induces catalyst sintering. Herein, we reported the synthesis of a binary palladium nanoalloy on a halloysite nanotube support (PdM@Hal). This one-pot synthesis procedure was performed *via* ultrasound-enhanced reduction of metal precursors in aqueous solution containing dispersed halloysite nanotubes, using NaBH_4_ as reducing agent. Transmission electron microscopy revealed that the synthesized PdM@Hal catalysts preserved the morphology of the pristine support after synthesis and calcination, with good dispersion of the catalyst on the surface of the support. Promoted metal-support interactions revealed enhanced catalytic performance, following the order PdNi > PdCo > Pd > PdCu, with activation energies of 68–94 kJ mol^−1^.

## Abstract 2

Bimetallic Pd-supported halloysite nanotubes revealed outstanding catalytic activity towards catalytic methane oxidation especially PdNi.

## Graphical abstract

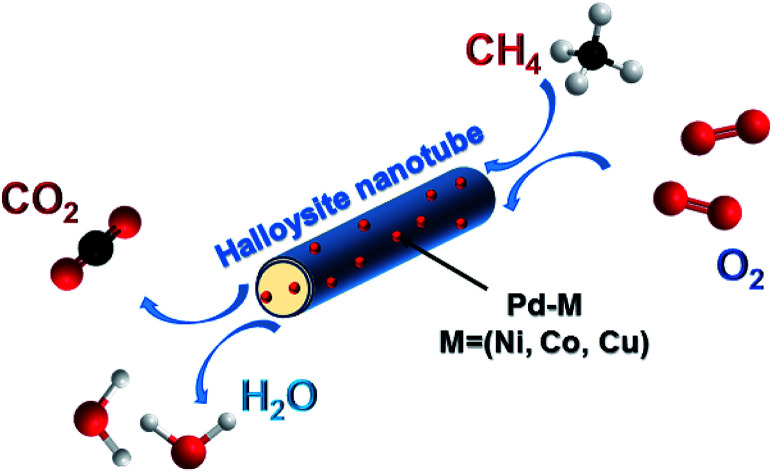

## Introduction

As a major constituent in natural gas, methane (CH_4_) is of great importance in multifarious applications, especially power generation. However, the escape of unburnt CH_4_ from exhaust gas streams during the combustion process has a deleterious effect on the environment, because methane as a greenhouse gas is 20 times as potent as CO_2_.^1^ Methane is an appealing fuel owing to the low concentrations of nitrogen and sulfur contaminants, along with the low CO_2_ emission per energy produced due to the high H/C ratio compared to other conventional fuels.^2,3^ However, because of its structural symmetry and relatively high chemical stability (*i.e.* it is the least reactive hydrocarbon^4^), the rate determining step for the oxidation reaction is the dissociative adsorption step (the activation and dissociation of the first C–H bond), which has a high energy barrier.^5^

To this end, great efforts have been devoted to developing efficient catalysts that can oxidize methane completely at low temperatures. Noble elements, *e.g.*, Pt,^6,7^ Ir,^8,9^ Ru,^10^ Pd,^11,12^ and Rh,^13,14^ have been recognized as highly reactive catalysts for methane oxidation.^15–17^ Among them, Pd has been reported to possess the highest catalytic activity towards methane oxidation, especially under oxidizing conditions.^18^ However, the deactivation of catalytic activity at high temperatures induced by water adsorption, the formation of Pd(OH)_2_, and sintering as a result of Ostwald ripening, are reported as the main obstacles hindering the use of Pd-based catalysts for methane oxidation. Several factors influence the oxidation mechanism, catalytic activity, and the resistance to deactivation of Pd-based catalysts, such as the type of support, particle size, catalyst-support interaction, oxidation state of the metal, and the dispersion of the catalyst.^17^

One of the main ways to improve the catalytic activity of Pd-based catalysts for methane oxidation is to alloy Pd with a second metal, such as Fe, Ni, Co or Cu, as a means of tuning the activity and stability of a Pd-based catalyst. Alloying with a second metal can tune the d-band center of Pd, the parameter that determines the capability of d-electrons to bind chemical species through electron donation or capture.^19–21^ This can ultimately influence the adsorption/activation of methane, thus altering the kinetics of the oxidation process.^22^ For instance, the poisoning effect of SO_2_ on the methane oxidation activity of PtPd/Al_2_O_3_ using various gas compositions has been investigated.^23^ The results revealed that the activity of bimetallic PtPd/Al_2_O_3_ was enhanced relative to the activity of monometallic Pd/Al_2_O_3_ and Pt/Al_2_O_3_. The enhanced activity of the bimetallic sample was attributed to the fact that alloying Pd with Pt enhanced the resistance to –OH formation and changed the oxidation mechanism compared to monometallic Pd under the same experimental conditions. In addition, bimetallic samples could withstand the deactivation by SO_2_ for prolonged periods compared with monometallic species.^23^ Likewise, methane oxidation activity has been studied on three-dimensionally ordered macroporous CeO_2_ loaded with Pd@Co nanoparticles, Co@Pd/3DOM CeO_2_, of different molar ratios in the range of 2.4–13.6 *via* a modified PVA-protected reduction procedure using polymethylmethacrylate as a template.^24^ Co_3.5_Pd exhibited the highest methane oxidation activity with *T*_90_% = 480 °C at SV = 40 000 mL g^−1^ h^−1^ and the lowest activation energy, *E*_a_ of 58 kJ mol^−1^. The boosted performance was ascribed to the enhanced oxygen and methane adsorbability and the unique properties of the core–shell structure.^24^

The nature of the support is considered to be one of the main factors affecting the catalytic activity. The role of the support is to provide a high surface area on which the active catalyst is dispersed, sufficient interaction with the catalyst to prevent its sintering, and may give rise to new active sites for the reaction at the support/catalyst interface.^25,26^ Various types of supports have been investigated for the methane oxidation reaction, such as metal oxides, *i.e.* Al_2_O_3_, ZrO_2_, CeO_2_, TiO_2_ and SiO_2_, zeolites,^27–30^ nitrogen-doped carbon,^31^ perovskites,^32,33^ and mesoporous silica supports.^34,35^ Halloysite is a naturally occurring aluminosilicate clay (Al_2_Si_2_O_5_(OH)_4_·*n*H_2_O), which has outstanding structural and chemical merits, such as high surface area, good chemical and thermal stabilities, and is inexpensive and environmentally friendly.^36^ Halloysite has a hollow tubular structure with nanometer dimensions and comprises a two-layered structure. Al–OH groups are present in the inner surface and Si–O groups dangle along the outer surface, which are inherent anchoring sites for various types of materials including polymers, drugs, metal oxides and metal nanoparticles.^37–40^ Despite the promising properties of halloysite as a potential host for different types of materials, its use as a support for heterogeneous catalysis is still rare^41–43^ and has not been reported for the methane oxidation reaction.

Inspired by this, herein we report a one-pot reduction method for the rational synthesis of binary metallic PdM@Hal with a small loading content of a Pd-based catalyst (<1 wt%). The synthesized PdM@Hal catalysts retained the one-dimensional nanotubular morphology of the pristine halloysite support along with its high surface area. Furthermore, they exhibited pronounced activity and stability towards methane oxidation, especially PdCo@Hal, which manifested the highest activity and stability compared to PdNi@Hal, PdCu@Hal and Pd@Hal.

## Experimental

### Materials

Halloysite nanoclay, potassium tetrachloropalladate (K_4_PdCl_4_), copper nitrate trihydrate (Cu(NO_3_)_2_·3H_2_O), cobalt nitrate hexahydrate (Co(NO_3_)_2_·6H_2_O), nickel nitrate hexahydrate (Ni(NO_3_)_2_·6H_2_O) and sodium borohydride (NaBH_4_) were purchased from Sigma-Aldrich Co. Ltd. All reagents were used without further purification.

### Synthesis of Pd-based catalysts

In a flask containing 500 mL deionized water, 100 milligrams of halloysite nanotubes were added and the mixture was ultrasonicated for 5 h. After this time, K_4_PdCl_4_ (5 mL, 20 mM) and MCl_2_·*x*H_2_O (3 mL, 20 mM) (M = Co or Ni or Cu) were added with continuous stirring for 2 h. After that, 5 mL of ice-cooled 0.1 M NaBH_4_ solution was added dropwise under ultrasound irradiation, and the final product was collected by centrifugation, washed with water and ethanol, and dried in a vacuum oven at 60 °C. The monopalladium counterpart (Pd@Hal) was synthesized using the same procedure, but with the addition of K_4_PdCl_4_ as the only metal precursor.

### Characterization

The morphology and composition of the as-synthesized materials were investigated using a Transmission Electron Microscope (TEM) (FEI Tecnai G2 TF20 UT) equipped with Energy Dispersive X-ray (EDX) spectroscopy and high-angle annular dark-field scanning TEM (HAADF-STEM) operated at 200 kV. The elemental composition of the as-synthesized catalysts was determined using Inductively Coupled Plasma-Mass Spectrometry (ICP-MS) on a NEXION 300D (PerkinElmer, USA). Textural properties of the as-synthesized catalysts were determined from N_2_ sorption experiments at 77 K. The surface area was estimated according to the BET (Brunauer–Emmett–Teller) model. The crystalline phases of the prepared materials were investigated using Powder X-ray Diffraction (PXRD) on an X'Pert-Pro MPD diffractometer (PANalytical Co., Netherlands) with a Cu-K_α_ source (*λ* = 1.54059 Å). The chemical nature and oxidation states of various elements were investigated *via* an XPS spectrophotometer (Kratos Axis Ultra) equipped with a monochromatic Al-K_α_ radiation source (1486.6 eV). A Micromeritics Autochem 2910 equipped with a thermal conductivity detector (TCD) was used to investigate H2-TPR. 100 mg of the test sample was introduced into a testing tube and 10% H_2_ in Ar was purged over the test sample at a flow rate of 30 mL min^−1^. The sample was then heated up to 850 °C at a heating rate of 5 °C min^−1^.

### Methane oxidation activity

Methane oxidation measurements were performed in a fixed-bed quartz reactor (id = 6 mm) at ambient pressure. 50 mg of each catalyst was placed at the center of the reactor and mounted with quartz wool. Prior to the activity measurements, the catalyst was oxidized in 20 wt% O_2_/Ar (50 mL min^−1^) at 500 °C for 2 h, where the temperature was increased at a constant heating rate of 5 °C min^−1^. The feed gas, comprising 1 wt% CH_4_ and 20 wt% O_2_ in Ar, was supplied at a rate of 60 mL min^−1^ (GHSV = 72 000 mL g^−1^ h^−1^), which was controlled by digital mass flow controllers. Gaseous products were analyzed using an infrared gas analyzer (IR200, Yokogawa, Japan) in order to estimate methane conversion. The reaction temperature was recorded *via* a K-type thermocouple located at the catalyst bed. Stability measurements were investigated at 350 °C under the continual flow of the reacting gases.

## Results and discussion

**Fig. 1:**
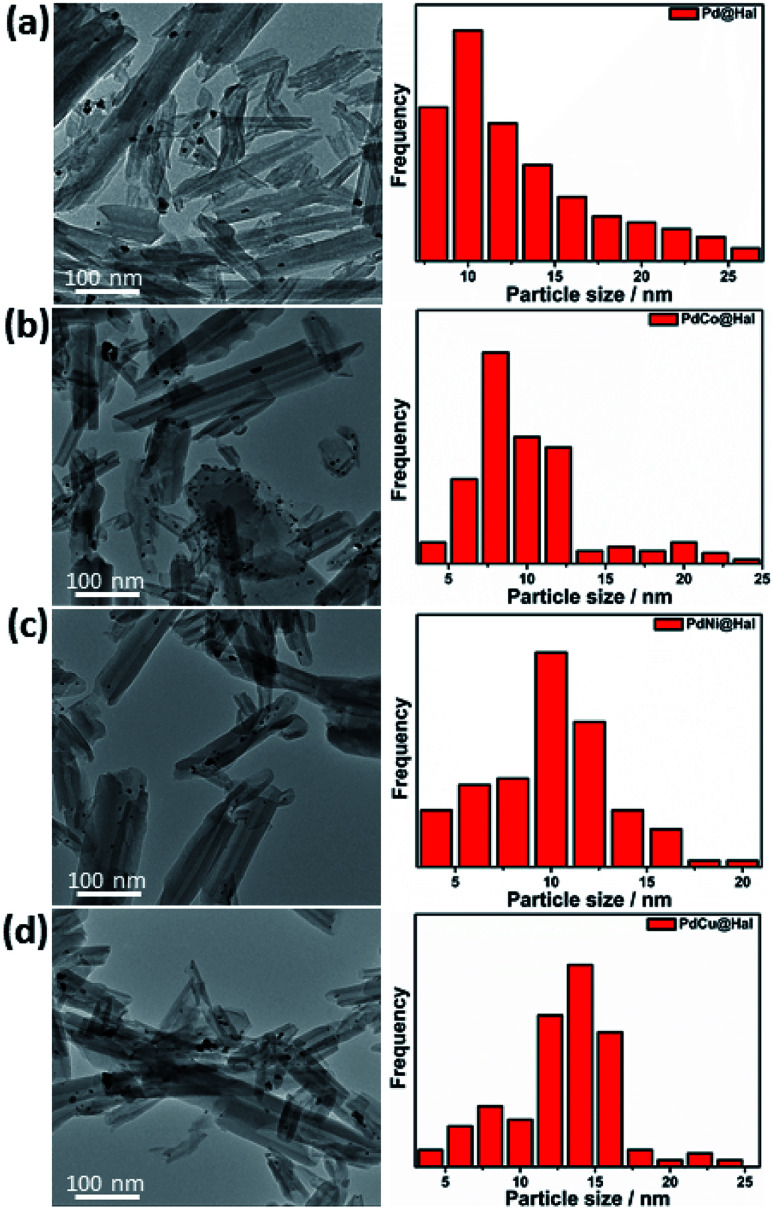
Low magnification TEM images and particle size distribution of (a) Pd, (b) PdCo, (c) PdNi, and (d) PdCu@Hal.

Monometallic and bimetallic palladium alloys were prepared on halloysite nanotubes *via* a single-step reduction process using NaBH_4_ as reductant followed by annealing of the formed products in H_2_ at 450 °C. TEM images of the monometallic and bimetallic Pd-based catalysts are presented in Fig. 1. The images show that all catalysts retained the nanotubular morphology of pristine halloysite nanotubes, along with deformed walls and edges owing to ultrasonication during the synthesis step. The images also revealed that the metal nanoparticles were mainly present at the outer surfaces of the halloysite nanotubes. The particle size distribution of the different Pd-based catalysts (as determined from TEM imaging) is presented in Fig. 1. The catalysts exhibited a relatively wide range of 4–25 nm, with average particle sizes of 7–9 nm.

**Fig. 2:**
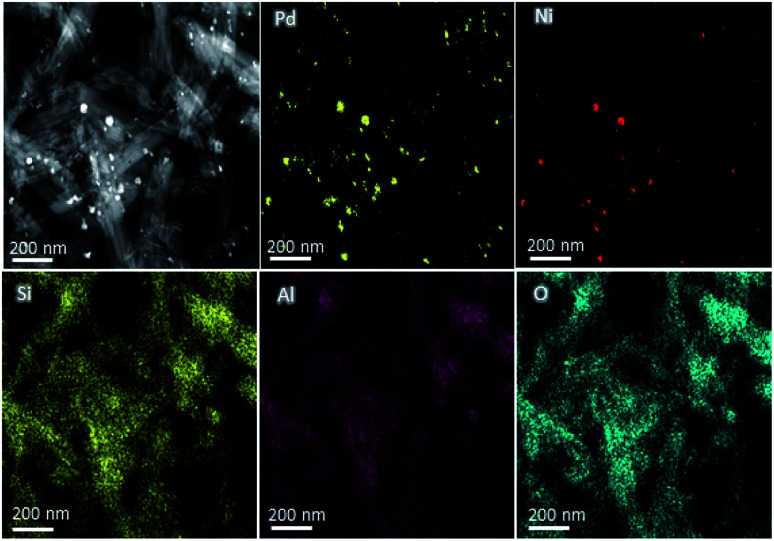
HAADF-EDS elemental mapping of PdNi@Hal.

The compositions of the Pd-based catalysts PdNi@Hal were investigated using HAADF-STEM analysis. Fig. 2 shows the elemental mapping of PdNi@Hal, which implies an even distribution of the different metals, with an atomic ratio of Pd to Ni of 3 : 1 as obtained from EDX analysis. The compositions of the different halloysite nanotube-based catalysts were further investigated using ICP-MS, and are given in Table 1. The compositions revealed that the average Pd loading is about 0.9 wt% for all catalysts and the average ratio of the Pd to the alloying metal is almost 3 : 1.

**Table 1:** The metallic composition of various Pd-based catalysts expressed in weight (%)

Catalyst	Pd	Co	Ni	Cu
PdNi@Hal	0.91	—	0.33	—
PdCo@Hal	0.90	0.32	—	—
PdCu@Hal	0.94	—	—	0.36
Pd@Hal	0.92	—	—	—

**Fig. 3:**
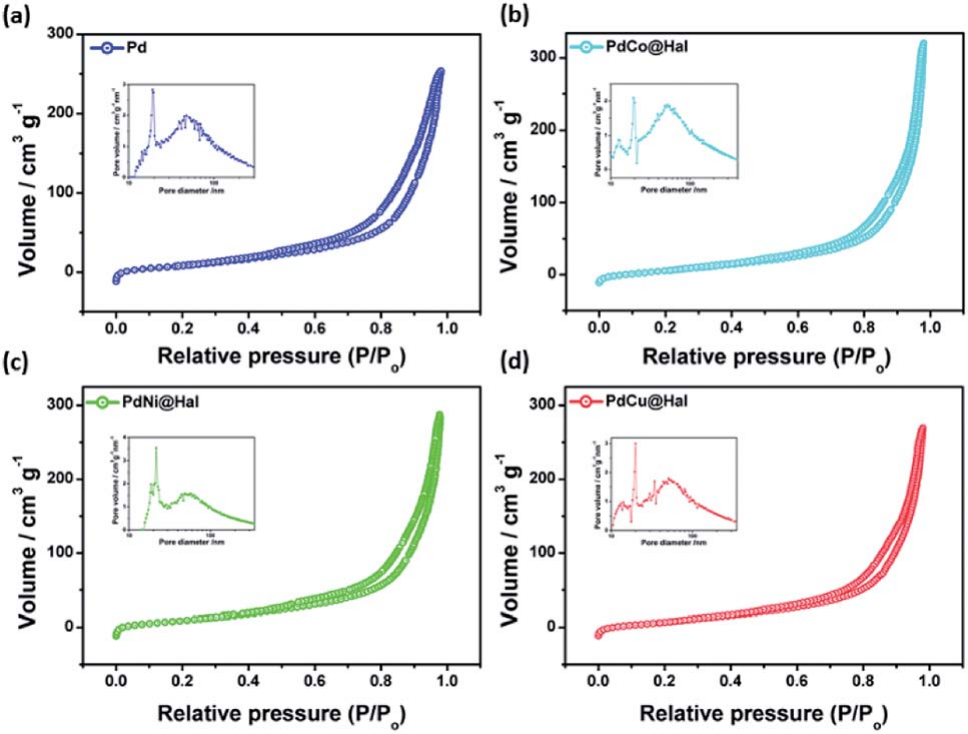
Adsorption–desorption isotherms and BJH pore size distributions (insets) of as-synthesized catalysts.

Fig. 3 shows nitrogen sorption isotherms and the pore size distributions of the as-synthesized catalysts. According to IUPAC nomenclature, all isotherms follow type IV isotherms.

All of the catalysts exhibit isotherms similar to halloysite nanotube support, indicating that the porous tubular structures were maintained after metal deposition. The specific surface areas and pore sizes were calculated from the corresponding BJH isotherms and are given in Table 2. The metal-loaded halloysite nanotubes exhibited surface areas of 86–90 m^2^ g^−1^, pore radii of 7–9 nm, and pore volumes of 0.43–0.51 cm^3^ g^−1^, which are slightly lower than those of pristine Hal (Fig. S1†) due to the partial blockage of pores with metallic nanoparticles. The reasonable surface area exhibited by all catalysts can afford more active sites for the catalytic oxidation reaction.

**Table 2:** The textural properties of the different Pd-based catalysts

Catalyst	Surface area (m^2^ g^−1^)	Average pore radius (nm)	Pore volume (cm^3^ g^−1^)
PdNi@Hal	85.6	8.3	0.51
PdCo@Hal	87.2	7.9	0.49
PdCu@Hal	88.9	8.6	0.48
Pd@Hal	89.6	7.4	0.43
Hal	93.4	9.1	0.54

**Fig. 4:**
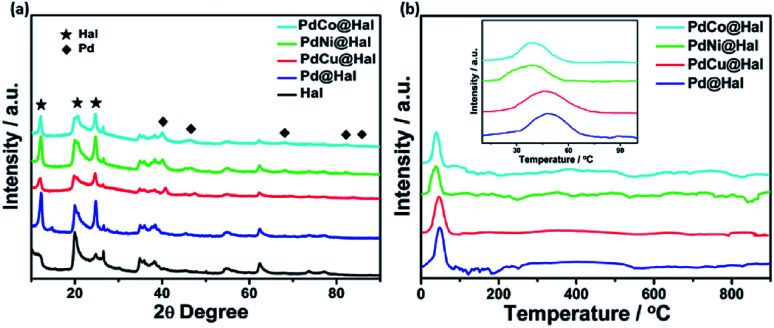
(a) XRD spectra and (b) H_2_-TPR profiles of the as-synthesized catalysts.

Fig. 4 shows the X-ray diffraction patterns of pristine halloysite nanotubes as well as the Pd-supported halloysite nanotubes. All diffraction peaks of the halloysite nanotubes are assigned to monoclinic halloysite (PDF 29-1487).^44,45^ For instance, the diffraction peak at 2*θ* = 12.1 is ascribed to the (001) diffraction recognized as halloysite-7 Å.^46^ Meanwhile, the diffractions noticed at 20.2 and 24.8 are ascribed to (020) and (002) planes of the halloysite nanotubes, respectively. In comparing the XRD pattern of the halloysite support to the metal-supported samples, it can be noticed that the Hal conserved its crystalline structure after the deposition of the Pd and PdM nanoalloys. Furthermore, the diffractions at 40, 47, 68, 82 and 87° can be assigned to the (111), (200), (220), (311) and (222) facets of the face-centered cubic structure of metallic Pd (JCPDS no. 46-1043), which implies crystallization of Pd over the halloysite nanotubes.^47^ The diffraction peaks of single metallic Ni, Co and Cu species were not detected, implying full integration of Ni, Co and Cu in the Pd lattice structure with the formation of bimetallic alloys with no observable undesirable phases. The diffractions of bimetallic Pd displayed slight shifts to higher 2*θ* values as compared to monometallic Pd, owing to the decrease in the lattice constants of the PdM nanoalloys compared to Pd, which can be attributed to the partial replacement of Pd atoms (of larger size) with Co, Ni or Cu atoms (of smaller size). This led to the contraction of the Pd lattice along with the modification of the Pd electronic structure.^48–51^

H_2_-TPR profiles of PdM@Hal are given in Fig. 4b. The results exhibit no reduction peaks for the halloysite support (Fig. S2†), which reveals high chemical stability. All catalyst profiles reveal one reduction peak located at around 45 °C, which can be assigned to the reduction of PdO. There were no observable peaks for the other metallic components, due to their low concentrations. The PdO reduction peak temperature follows the order PdNi < PdCo < PdCu < Pd@Hal, affirming the easier PdO reducibility over PdNi@Hal compared to the other catalysts. The interaction of Pd with Ni, Co and Cu is accompanied by charge transfer from these metals to Pd, which facilitates the reduction of PdO and shifts the reduction peak to lower temperatures compared to pure Pd.^52^ Despite the existence of hydroxyl groups in the halloysite support, which are capable of interacting with various metal particles, the recorded low reduction temperature of PdO verifies the weak interaction between the metallic nanoparticles and the support material. This implies that the metallic nanoparticles are mainly located at the outer surfaces of the support where only Si–O groups are available which do not efficiently interact with the catalyst.

**Fig. 5:**
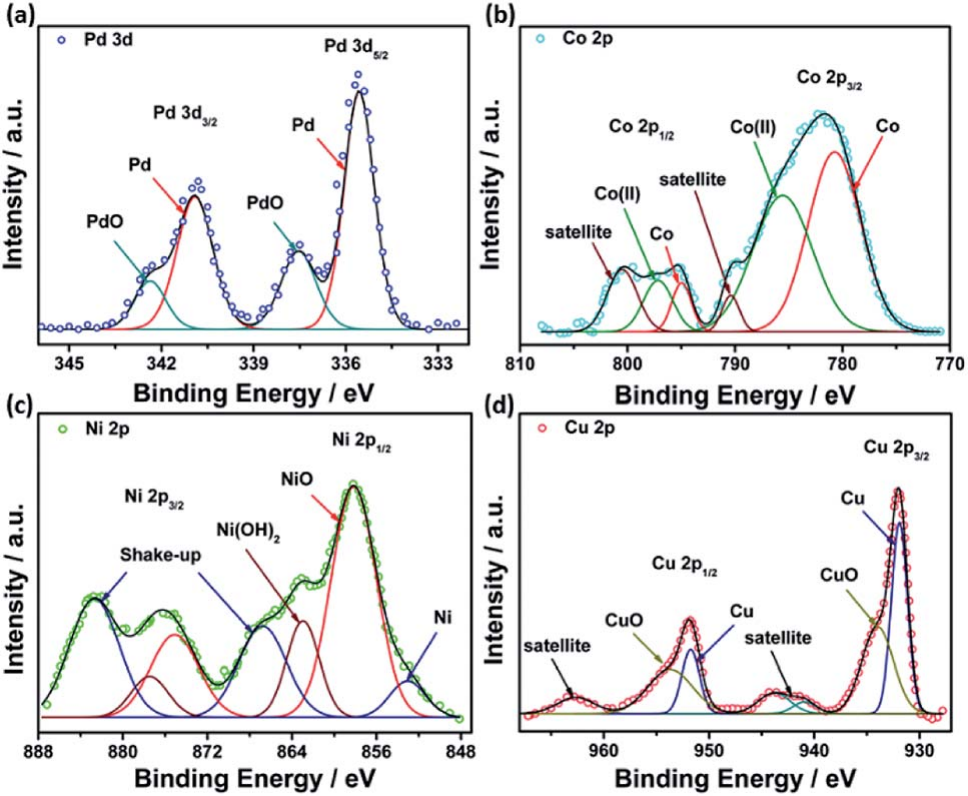
XPS high resolution spectra of (a) Pd 3d, (b) Co 2p, (c) Ni 2p, and (d) Cu 2p.

XPS analysis was performed to investigate the chemical composition and valence states of different elements in the studied catalysts. Deconvolution of Pd(3d) in all catalysts revealed two peaks at around 335.3 and 340.8 eV, assigned to Pd(0), while the other two peaks observed at 337.0 and 342.0 eV are ascribed to Pd(ii) oxide (Fig. 5a). A high-resolution spectrum of Co 2p revealed the existence of two peaks at 780.7 and 794.9 eV assigned to metallic Co, while those observed at 785.5 and 496.9 eV are attributed to Co(ii) oxide^53^ (Fig. 5b). Similarly, PdNi@Hal displayed two peaks in the Ni 2p region at 851.5 and 856.5 eV, which can be attributed to metallic Ni(0) and Ni(ii), respectively, whereas the peaks located at 861.5 and 880.9 eV are shake-up peaks originating from multi-electron excitations^54^ (Fig. 5c). The predominant species of Ni are the oxidized species, which can be ascribed to the oxophilic nature of Ni surface atoms, which rapidly react with atmospheric oxygen or H_2_O to form oxidized species, *i.e.* NiO and Ni(OH)_2_.^54,55^ Two peaks were observed in the Cu 2p region at 932.0 and 951.8 eV, assigned to Cu 2p_3/2_ and Cu 2p_1/2_, whereas the other two peaks observed at the higher binding energy side (942.6 and 963.0 eV) are satellite peaks encountered by the presence of unfilled 3d.^9,56^ Deconvolution of the Cu 2p peaks revealed two peaks at 931.9 and 951.7 eV, assigned to metallic Cu, and lower intensity peaks at 934.2 and 953.7 eV, corresponding to CuO (Fig. 5d).^57^

The Pd 3d peaks are shifted to higher binding energies in the bimetallic catalysts compared to Pd@Hal (Fig. S3†). This is attributed to the interaction between Pd and Ni, Co and Cu, induced by electron transfer between Pd and the other metals, which alters the electronic structure of Pd, modifies its d-band center, and enhances catalytic performance.^58^

**Fig. 6:**
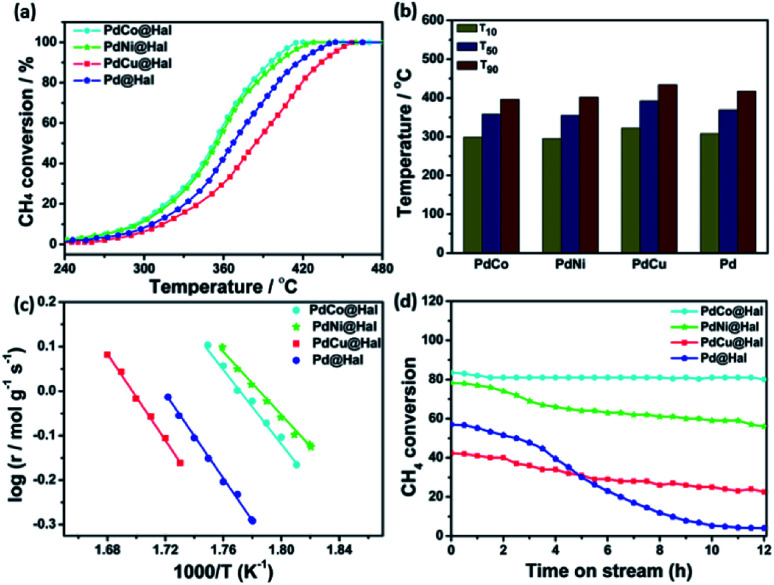
(a) Light-off methane oxidation curves. (b) Temperatures of 10, 50, and 90% conversions. (c) Arrhenius plots. (d) Stability tests of monometallic and bimetallic Pd-catalysts at 380 °C.

The catalytic activity of methane oxidation over different Pd-based catalysts was investigated using a fixed-bed reactor at atmospheric pressure. The reactivity of the samples was expressed in terms of methane conversion (%) as a function of temperature (Fig. 6a). For all of the catalysts, the methane conversion increased with rising the temperature until it reached 100% conversion. For all investigated Pd-based catalysts, CO was not detected in the outlet stream, which confirms that methane was completely oxidized to CO_2_ (*i.e.* CH_4_ + 2O_2_ → CO_2_ + 2H_2_O). All bimetallic catalysts exhibited higher activity towards methane oxidation than the monometallic Pd counterpart.

Amongst all of the catalysts, PdCo expressed the highest methane oxidation activity. For instance, methane conversion reached 10% at 295 °C over PdNi, while it reached the same conversion at 299, 308 and 322 °C, in the case of PdCo, Pd and PdCu, respectively. Similarly, the temperature for 50% conversion (*T*_50_) was 355 °C in the case of PdNi, which is 3, 14 and 37 °C, lower than that of PdCo, Pd and PdCu, respectively. Furthermore, complete methane conversion was attained over PdNi at 428 °C; however, it was reached at 420, 444, and 465 °C in the case of PdCo, Pd and PdCu, respectively (Fig. 6b). The data obtained revealed that the activity towards methane oxidation follows the order: PdNi > PdCo > Pd > PdCu.

The rates of methane combustion and the analogous turnover frequencies of the as-synthesized Pd-based catalysts are given in Table 3. Measurements were performed at low rates of conversion (below 20%) to lessen the diffusion effect. Monometallic and bimetallic Pd are the active species for methane oxidation, hence, all calculations were done on their basis. The calculated reaction rate on PdNi@Hal was 4.01 × 10^−2^ mol g^−1^ s^−1^ at 250 °C, which is 3.8, 1.5 and 1.9 times higher than those for PdCu, PdCo and Pd@Hal, respectively. Furthermore, the turnover frequency of the various investigated catalysts estimated at 250 °C followed the order: PdNi (0.32 s^−1^) > PdCo (0.24 s^−1^) > Pd (0.21 s^−1^) > PdCu@Hal (0.11 s^−1^).

**Table 3:** Kinetic parameters over Pd-based catalysts at 250 °C

Catalyst	*r* × 10^−2^ (mol g_Pd_^−1^ s^−1^)	Particle size (nm)	*D* (%)	TOF (s^−1^)	*E*_a_ (kJ mol^−1^)
PdNi@Hal	4.01	9.4	13.3	0.32	68
PdCo@Hal	2.74	10.2	12.3	0.24	80
PdCu@Hal	1.05	11.8	10.6	0.11	94
Pd@Hal	2.07	12.1	10.3	0.21	91

The apparent activation energies for methane oxidation over monometallic and bimetallic Pd catalysts were estimated *via* the corresponding Arrhenius plots (Fig. 6c). Calculations were based on data collected at CH_4_ conversions of less than 10% to minimize mass transfer limitations. The estimated activation energies were found to be 68, 80, 94 and 91 kJ mol^−1^ for PdNi, PdCo, PdCu and Pd, respectively. PdNi and PdCo expressed lower activation energies compared to PdCu and Pd, which confirms the enhanced oxidation kinetics.

The stability of different catalysts was examined at 380 °C for a gas mixture containing 1 wt% methane at a space velocity GHSV of 72 000 h^−1^ over a period of 12 h (Fig. 6d). PdCo and PdNi@Hal exhibited enhanced stability compared to PdCu and Pd@Hal. Unlike the bimetallic catalysts, monometallic Pd expressed a higher rate of activity decay with time. This confirms the role of alloying in the stabilization of Pd.

The mechanism of oxidation of methane on Pd-based catalysts is still a subject of debate. Some studies report that metallic Pd is the surface active phase,^59^ whereas other studies claim that PdO is the active phase for this reaction.^60^ However, at high temperatures (specially above 600 °C) PdO is reduced to Pd even under oxidizing conditions, which leads to catalyst deactivation.^61,62^ Hence, it has been argued that the highest catalytic activity is obtained when a mixed phase of Pd/PdO is available.^63,64^ It has been reported that the reaction proceeds initially *via* adsorption and dissociation of methane to methyl and hydrogen radicals (which is considered to be the rate determining step).^5^ This step takes place preferentially on metallic Pd; then, it is followed by subsequent oxidation by the adsorbed oxide species. Regardless of the nature of the active phase in the Pd-based catalyst, the activity and stability of the catalyst towards CCM is dependent on several factors, such as the crystallite size, specific surface area, nature of support, and Pd content on the surface. Among them, alloying with a second metal can greatly affect the catalytic performance of Pd-based catalysts. The impact of Pd alloying on methane combustion has rarely been studied and remains a subject of dispute. Willis *et al.*^22^ studied the impact of different bimetallic Pd nanocrystals on the CCM. Their study revealed that most PdM/Al_2_O_3_ catalysts have similar *T*_100_, *i.e.* 400 °C, and similar activation energies (78–87 kJ mol^−1^); however, the alloying elements enhanced the rate of oxidation and turnover frequency (TOF), especially in the case of Ni and Zn. Additionally, the thermal stabilities of catalysts were enhanced in the case of Fe, Co, Ni and Zn, as revealed by the improvement of the redox properties of the PdO active phase. The enhancement of stability was attributed to inhibition of PdO sintering in the case of Fe, Co and Sn, and an increase of PdO thermal stability in the case of Ni and Zn.^22^ The catalytic activity of different bimetallic Pd alloys, *i.e.* PdPt, PdIr, PdNi, PdCo, PdCu, PdAu, PdAg, and PdRh, revealed similar or lower CCM activity compared to monometallic Pd, and only a slight improvement in the catalyst stability was observed in the case of PdPt and PdAg.^65^ In contrast, it was reported that NiO enhanced the activity of Pd/Al_2_O_3_, owing to the suppression of PdO decomposition at high temperatures.^66^ According to the results obtained here, it seems that Ni and Co oxides have a positive effect on the activity of PdO, whereas Cu oxide exhibits a deactivating effect on PdO.

Methane oxidation over Pd-based catalysts takes place *via* dissociation of CH_4_ at the vacancy/oxide active sites, yielding the formation of adsorbed methyl radicals and surface hydroxyl groups (CH_4_ + Pd*/PdO = Pd–CH_3_ + Pd–OH).^71,72^ This step is followed by recombination of surface hydroxyls to form H_2_O, PdO and surface vacancies (Pd–OH + Pd–OH = Pd–O + H_2_O + Pd*). Pd–O is regenerated by combining of vacancies to O_2_ (2Pd* + O_2_ = 2Pd–O) and the regenerated Pd–O can abstract H from methyl radicals for further oxidation.^71,72^ The rate of methane oxidation is dependent on the rate of O_2_ activation, in addition to the rate of formation of hydroxyl species. Ni and Co are recognized for their oxophilic nature as well as high oxygen storage capacity, so they can stabilize PdO and enhance the activation of O_2_.^54,73^ This can explain the positive effect of their oxides in the methane oxidation reaction. In the case of Cu, the low oxidation activity can be assigned to the partial coverage of Pd and PdO with CuO, which decreases the number of active sites participating in the oxidation reaction.^74^ Furthermore, Cu has inferior reactivity towards O_2_ activation compared to Co and Ni, which may slow down the oxidation kinetics.^58,75^

Compared to metal oxide supports, halloysite nanotubes have a great captivating nature compared to traditional metal oxides and a great ability to confine and stabilize metallic nanoparticles and prevent their sintering. Furthermore, the halloysite support has a greater hydrothermal stability than oxides, especially at low temperatures, and hence has a reduced poisoning effect on the methane oxidation activity. Such a poisoning effect can originate due to water absorption and accumulation of hydroxyl groups on the surface, which retards the activation of methane molecules and enhances steam-induced sintering. Comparing the catalytic activity of PdM@Hal to that of previously reported catalysts confirms the promising performance of halloysite nanotubes as a support for the methane oxidation reaction (Table 4). These results will pave the way for the wide scale application of halloysite nanotube supports in heterogeneous catalysis, after controlled surface modifications to tune the hydrophilicity, acidity and interaction with metallic catalysts.

**Table 4:** Comparison of PdNi@Hal activity towards methane oxidation with reported catalysts in literature

Catalyst	Metal loading (wt%)	WHSV (mL g^−1^ h^−1^)	*T*_10_ (°C)	*T*_50_ (°C)	*T*_90_ (°C)	*E*_a_ (kJ mol^−1^)	Reference
Co_3.5_Pd/three dimensionally ordered macroporous CeO_2_	0.26	40 000	356	430	480	58	24
Flame made Pd/TiO_2_ nanoparticles	5	—	305	350	>500	—	67
NiO@PdO/Al_2_O_3_	0.2	30 000	255	318	370	89	68
La(Fe,Pd)O_3_	2.28		>400	545	565	—	69
La(Mn,Pd)O_3_	2.32	60 000	>400	524	550	—	
PdO@CeO_2_/mesoporous silica	1	180 000	∼250	290	342	—	70
	5		∼300	350	442	—	
Pd/CeO_2_	0.81	200 000	291	∼350	∼490	—	11
PdNi/halloysite nanotubes	0.9	72 000	295	355	402	68	This work

## Conclusions

Bimetallic palladium alloys/halloysite nanotubes were prepared *via* the *in situ* reduction of the metal ion precursors on the surface of halloysite using NaBH_4_. The metallic nanoparticles were attached to the outer surface of the halloysite support and retained their tubular structure with a Pd metallic loading of 0.9 wt% and an average particle size of 7–9 nm. All of the studied Pd-based catalysts revealed high methane oxidation activity following the order PdNi > PdCo > Pd > PdCu, with activation energies of 68–94 kJ mol^−1^. Related future plans (already initiated) include the synthesis and characterization of modified halloysite nanotubes using different chemical treatments and investigating the effect of the treatment approaches on the catalytic performance of Pd-supported halloysite nanoclay for methane oxidation.

## Conflicts of interest

There are no conflicts to declare.

## Supplementary Material

RA-009-C9RA06804J-s001

## References

[cit1] Le Mer J., Roger P. (2001). Eur. J. Soil Biol..

[cit2] Karavalakis G., Durbin T. D., Villela M., Miller J. W. (2001). J. Nat. Gas Sci. Eng..

[cit3] Fouladvand S., Skoglundh M., Carlsson P. A. (2014). Catal. Sci. Technol..

[cit4] Yoon J., Lim Y., Choi B., Hwang H. (2014). Int. J. Hydrogen Energy.

[cit5] Burch R., Hayes M. (1995). J. Mol. Catal. A: Chem..

[cit6] Aghalayam P., Park Y. K., Fernandes N., Papavassiliou V., Mhadeshwar A., Vlachos D. G. (2003). J. Catal..

[cit7] Korup O., Goldsmith C. F., Weinberg G., Geske M., Kandemir T., Schlögl R., Horn R. (2013). J. Catal..

[cit8] Nakagawa K., Anzai K., Matsui N., Ikenaga N., Suzuki T., Teng Y., Kobayashi T., Haruta M. (1998). Catal. Lett..

[cit9] Basini L., Aragno A., Vlaic G. (1996). Catal. Lett..

[cit10] Lanza R., Järås S. G., Canu P. (2007). Appl. Catal., A.

[cit11] Danielis M., Colussi S., de leitenburg C., Soler L., Llorca J., Trovarelli A. (2018). Angew. Chem., Int. Ed..

[cit12] Grunwaldt J.-D., van Vegten N., Baiker A. (2007). Chem. Commun..

[cit13] Rice S. F., McDaniel A. H., Hecht E. S., Hardy A. J. (2007). Ind. Eng. Chem. Res..

[cit14] Shan J., Li M., Allard L. F., Lee S., Flytzani-Stephanopoulos M. (2017). Nature.

[cit15] Gélin P., Primet M. (2002). Appl. Catal., B.

[cit16] Lin W., Zhu Y., Wu N., Xie Y., Murwani I., Kemnitz E. (2004). Appl. Catal., B.

[cit17] Yoshida H., Nakajima T., Yazawa Y., Hattori T. (2007). Appl. Catal., B.

[cit18] Burch R., Urbano F., Loader P. (1995). Appl. Catal., A.

[cit19] Ruban A., Hammer B., Stoltze P., Skriver H. L., Nørskov J. K. (1997). J. Mol. Catal. A: Chem..

[cit20] Gunji T., Noh S. H., Ando F., Tanabe T., Han B., Ohsaka T., Matsumoto F. (2018). J. Mater. Chem. A.

[cit21] Sha Y., Yu T. H., Merinov B. V., Goddard III W. A. (2014). ACS Catal..

[cit22] Willis J. J., Goodman E. D., Wu L., Riscoe A. R., Martins P., Tassone C. J., Cargnello M. (2017). J. Am. Chem. Soc..

[cit23] Sadokhina N., Smedler G., Nylén U., Olofsson M., Olsson L. (2018). Appl. Catal., B.

[cit24] Xie S., Liu Y., Deng J., Zhao X., Yang J., Zhang K., Han Z., Dai H. (2016). J. Catal..

[cit25] Heck R. M. , Farrauto R. J. and Gulati S. T., Diesel engine emissions, Catalytic Air Pollution Control, John Wiley & Sons, 2009, pp. 238–294

[cit26] Kylhammar L., Carlsson P.-A., Skoglundh M. (2011). J. Catal..

[cit27] Lou Y., Ma J., Hu W., Dai Q., Wang L., Zhan W., Guo Y., Cao X.-M., Guo Y., Hu P. (2016). ACS Catal..

[cit28] Petrov A. W., Ferri D., Tarik M., Kröcher O., Van Bokhoven J. A. (2017). Top. Catal..

[cit29] Hammond C., Dimitratos N., Jenkins R. L., Lopez-Sanchez J. A., Kondrat S. A., Hasbi ab Rahim M., Forde M. M., Thetford A., Taylor S. H., Hagen H. (2013). ACS Catal..

[cit30] Petrov A. W., Ferri D., Krumeich F., Nachtegaal M., van Bokhoven J. A., Kröcher O. (2018). Nat. Commun..

[cit31] Soorholtz M., White R. J., Zimmermann T., Titirici M.-M., Antonietti M., Palkovits R., Schüth F. (2013). Chem. Commun..

[cit32] Lu Y., Keav S., Marchionni V., Chiarello G. L., Pappacena A., Di Michiel M., Newton M. A., Weidenkaff A., Ferri D. (2014). Catal. Sci. Technol..

[cit33] Huang Q., Li W., Lei Y., Guan S., Zheng X., Pan Y., Wen W., Zhu J., Zhang H., Lin Q. (2018). Catal. Lett..

[cit34] Gannouni A., Albela B., Zina M. S., Bonneviot L. (2013). Appl. Catal., A.

[cit35] Zribi S., Albela B., Bonneviot L., Zina M. S. (2015). Appl. Catal., A.

[cit36] Wang K., Zhang Y., Zhao J., Yan C., Wei Y., Meng M., Dai X., Li C., Yan Y. (2018). New J. Chem..

[cit37] Levis S., Deasy P. (2002). Int. J. Pharm..

[cit38] Shchukin D. G., Sukhorukov G. B., Price R. R., Lvov Y. M. (2005). Small.

[cit39] Singh B., Gilkes R. (1992). Clays Clay Miner..

[cit40] De Silva R. T., Dissanayake R. K., Mantilaka M. P. G., Wijesinghe S., Kaleel S. S., Premachandra T. N., Weerasinghe L., Amaratunge G. A., De Silva K. N. (2018). ACS Appl. Mater.
Interfaces.

[cit41] Barrientos-Ramírez S., Ramos-Fernández E., Silvestre-Albero J., Sepúlveda-Escribano A., Pastor-Blas M., González-Montiel A. (2009). Microporous Mesoporous Mater..

[cit42] Liu P., Zhao M. (2009). Appl. Surf. Sci..

[cit43] Machado G. S., de Freitas Castro K. A. D., Wypych F., Nakagaki S. (2008). J. Mol. Catal. A: Chem..

[cit44] Wang R., Jiang G., Ding Y., Wang Y., Sun X., Wang X., Chen W. (2011). ACS Appl. Mater. Interfaces.

[cit45] Zhang Y., Yang H. (2012). Phys. Chem. Miner..

[cit46] Brown G. and Brindley G. W., Crystal Structure of Clay Minerals and their X-ray Identification, Mineralogical Society London*,* 1980

[cit47] Yan Z., Hu Z., Chen C., Meng H., Shen P. K., Ji H., Meng Y. (2010). J. Power Sources.

[cit48] Ren X., Huang M., Luo S., Li Y., Deng L., Mi H., Sun L., Zhang P. (2018). J. Mater. Chem. A.

[cit49] Zhu F., Ma G., Bai Z., Hang R., Tang B., Zhang Z., Wang X. (2013). J. Power Sources.

[cit50] Rezaei M., Tabaian S. H., Haghshenas D. F. (2014). J. Mater. Chem. A.

[cit51] Cai B., Wen D., Liu W., Herrmann A. K., Benad A., Eychmüller A. (2015). Angew. Chem., Int. Ed..

[cit52] Liu C., Nan C., Fan G., Yang L., Li F. (2017). Mol. Catal..

[cit53] Tian Q., Chen W., Wu Y. (2015). J. Electrochem. Soc..

[cit54] Ahmad Y. H., Mohamed A. T., Youssef K. M., Kundu S., Mkhoyan K. A., Al-Qaradawi S. Y. (2019). Electrochem. Commun..

[cit55] Shen S., Zhao T., Xu J., Li Y. (2010). J. Power Sources.

[cit56] Li Z., Xin Y., Zhang Z., Wu H., Wang P. (2015). Sci. Rep..

[cit57] Jin Z., Liu C., Qi K., Cui X. (2017). Sci. Rep..

[cit58] Ahmad Y. H., Mohamed A. T., Hassan W. M. I., Soliman A., Mahmoud K. A., Aljaber A. S., Al-Qaradawi S. Y. (2019). Appl. Surf. Sci..

[cit59] Oh S. H., Mitchell P. J., Siewert R. M. (1991). J. Catal..

[cit60] Burch R., Urbano F. (1995). Appl. Catal., A.

[cit61] Farrauto R. J., Lampert J. K., Hobson M. C., Waterman E. M. (1995). Appl. Catal., B.

[cit62] Castellazzi P., Groppi G., Forzatti P. (2010). Appl. Catal., B.

[cit63] Farrauto R. J., Hobson M., Kennelly T., Waterman E. (1992). Appl. Catal., A.

[cit64] Epling W. S., Hoflund G. B. (1999). J. Catal..

[cit65] Persson K., Ersson A., Jansson K., Iverlund N., Järås S. (2005). J. Catal..

[cit66] Widjaja H., Sekizawa K., Eguchi K., Arai H. (1999). Catal. Today.

[cit67] Niu F., Li S., Zong Y., Yao Q. (2014). J. Phys. Chem. C.

[cit68] Zou X., Rui Z., Ji H. (2017). ACS Catal..

[cit69] Lu Y., Eyssler A., Otal E., Matam S., Brunko O., Weidenkaff A., Ferri D. (2013). Catal. Today.

[cit70] Dai Y., Kumar V. P., Zhu C., MacLachlan M. J., Smith K. J., Wolf M. O. (2017). ACS Appl. Mater. Interfaces.

[cit71] Zou X., Rui Z., Ji H. (2017). ACS Catal..

[cit72] Schwartz W. R., Ciuparu D., Pfefferle L. D. (2012). J. Phys. Chem. C.

[cit73] Stefanov P., Todorova S., Naydenov A., Tzaneva B., Kolev H., Atanasova G., Stoyanova D., Karakirova Y., Aleksieva K. (2015). Chem. Eng. J..

[cit74] Persson K., Ersson A., Jansson K., Iverlund N., Järås S. (2005). J. Catal..

[cit75] Ryu C. K., Ryoo M. W., Ryu I. S., Kang S. K. (1999). Catal. Today.

