# Supplementary material for: Natural clay-supported palladium catalysts for methane oxidation reaction: effect of alloying
Source: RSC Adv. 2019 Oct 15;9(56):32928–35. doi: 10.1039/c9ra06804j (PMC9073133; doi:10.1039/c9ra06804j)
Supplement: RA-009-C9RA06804J-s001 [file RA-009-C9RA06804J-s001.pdf]

Electronic Supplementary Material (ESI) for RSC Advances.
This journal is © The Royal Society of Chemistry 2019

Natural Clay-Supported Palladium Catalysts for Methane Oxidation Reaction: Effect of Alloying

Yahia H. Ahmad,[a] Assem T. Mohamed,[a] Khaled A. Mahmoud,[b] Amina S. Aljaber,[a] and Siham Y. Al-Qaradawi*,[a]

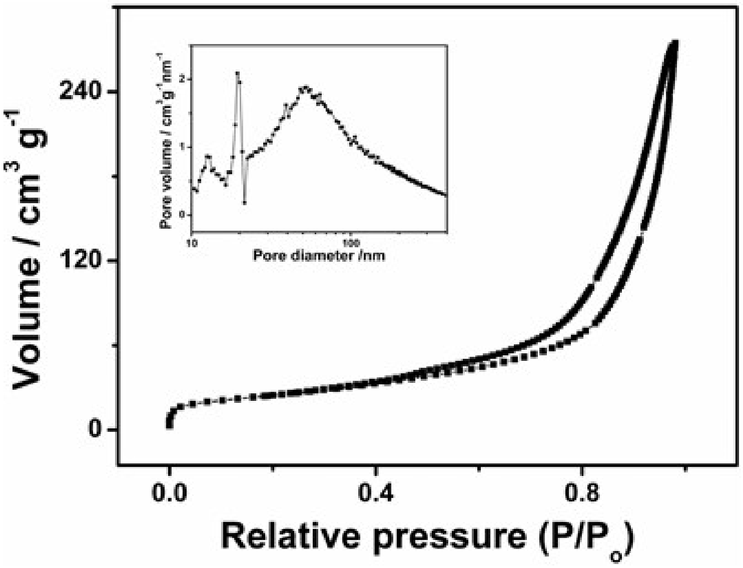

Fig. S1. N_2 Adsorption-desorption isotherms over pristine halloysite nanotubes

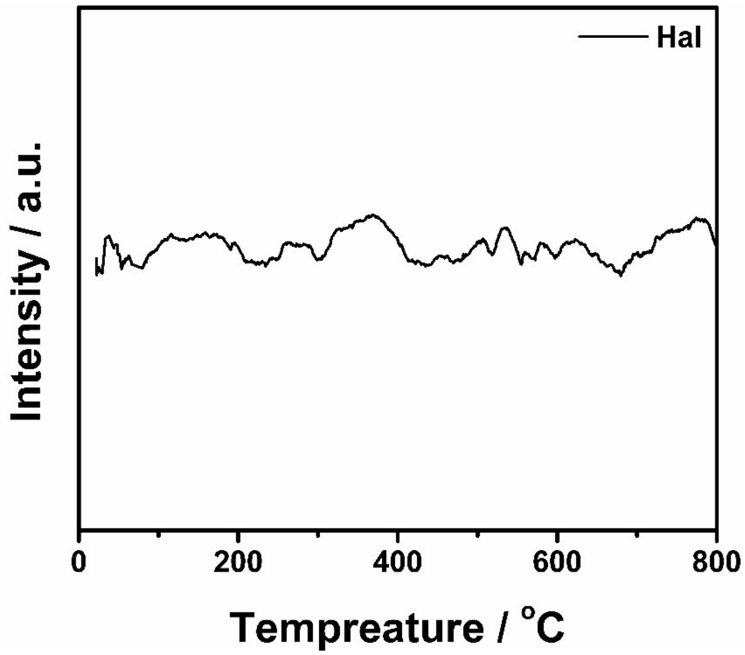

Fig. S2. H_2-TPR profile of halloysite nanotubes

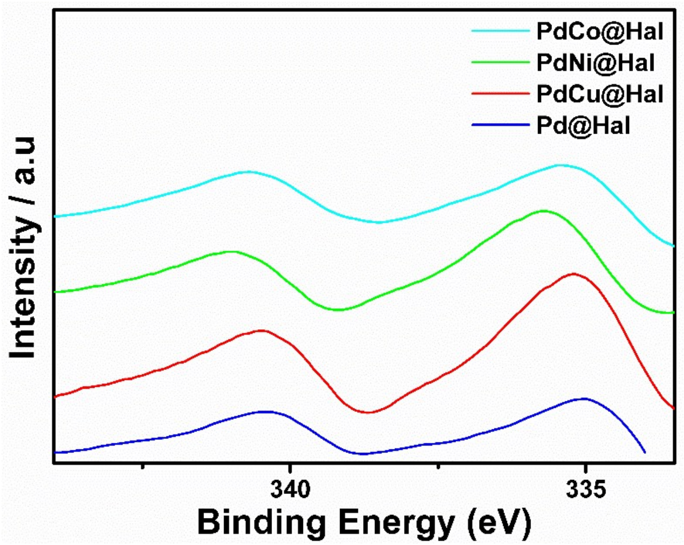

Fig. S3. High resolution XPS spectra of Pd 3d in different as-synthesized catalysts
